# Application of nanoparticles for salinity stress management and biofortification in wheat: a review of dual approaches and insights

**DOI:** 10.3389/fpls.2025.1592866

**Published:** 2025-07-04

**Authors:** Abhishek Singh, Roland Bol, Viktoriia Lovynska, Rupesh Kumar Singh, João Ricardo Sousa, Karen Ghazaryan

**Affiliations:** ^1^ Faculty of Biology, Yerevan State University, Yerevan, Armenia; ^2^ Institute of Bio- and Geosciences (IBG), Forschungszentrum Jülich, Jülich, Germany; ^3^ Centre for the Research and Technology of Agro-Environmental and Biological Sciences (CITAB), Inov4Agro, University of Trás-os-Montes and Alto Douro (UTAD), Vila Real, Portugal

**Keywords:** salinity stress, wheat, nanofortification, nanoparticles, food security

## Abstract

Salinity stress is one of the most challenging constraints affecting wheat production, limiting both yield and nutritional quality. Wheat is one of the most important staple cereals as well as a major source of carbohydrates for a considerable portion of the world population, yet wheat has suffered from significant productivity constraints due to salt stress. Such stress adversely affects germination, vegetative growth, reproductive organ development, enzymatic activity, photosynthesis photostability, and hormonal equilibrium, eventually causing oxidative stress and drastic loss of crop yield. Furthermore, the reducing nutritional quality of wheat further aggravates the issues regarding malnutrition and food security, highlighting the need for effective mitigation strategies. Although various methods have been investigated, including plant breeding, genetic engineering, and agronomic management, they are labor, cost, and time-intensive. Nanotechnology is a novel, eco-friendly and efficient approach for controlling salinity stress and improving crop biofortification. Some common methods of applications of nanotechnology-based products like nanoparticles (NPs) are foliar spraying, soil amendments and seed priming, which have shown considerable promise in improving salinity stress resistance, nutrient absorption, and wheat yield. This review outlines the extent of contribution of NPs in alleviating salinity stress, as well as the enhancement of the nutritional qualities of wheat. This work uniquely combines both salinity stress adaptation and nanofortification strategies under one framework that filling crucial information gaps. Investigating the mechanisms underlying NPs interaction with plant systems is essential for designing effective, green, and cost-efficient nanotechnology tools for sustainable wheat production. In the long run, this knowledge will aid sustainable agricultural practices and food security worldwide.

## 1 Introduction

As a crop, wheat is versatile, produces a lot, and is easy to store. These and other beneficial traits have been selectively enhanced by human societies, from the earliest forms of wheat to the varieties grown today (Venske et al., 2019). It was first domesticated approximately 10,000 years ago in the Fertile Crescent, and since then, early farmers have introduced it throughout the world by adjusting populations to suit different temperatures (Venske et al., 2019). Wheat crops have immense genetic diversity and can thrive in a wide range of climates, including temperate, Mediterranean, and subtropical zones, across both the Northern and Southern Hemispheres. For instance, *Triticum aestivum* L. has over 25,000 different varieties, each tailored to a certain temperate zone (Shewry, 2018). The timing of sowing determines the wheat variety. Sowing winter wheat in autumn allows the seedlings to experience temperatures of 0–5°C during their vegetative phase. As much as 80% of the wheat grown globally is winter wheat. South Asian and North African countries cultivate spring wheat in the spring and harvest it in late summer or autumn (Curtis et al., 2002). Different genomic compositions, grain compositions, and end uses can be found in modern wheat cultivars (*Triticum durum*), tetraploid durum wheat (*Triticum aestivum* L.), and hexaploid bread wheat. The rachis and glumes of these species were reorganized during domestication, transforming wild wheat into cultivated varieties. This morphological change has facilitated wheat cropping, thereby increasing its economic significance. Wheat seeds provide both carbohydrates and proteins, with endosperm proteins, prolamins, divided into gliadins and glutenin. The elasticity and extensibility of dough in breadmaking are primarily affected by the concentration of gliadin and glutenin. Wheat dough is unique in its processing into various types of bread, bakery products, pasta, and other processed foods. Wheat cultivars with certain quality qualities are essential to markets and processing companies, such as grain protein content and hardness.

Soils affected by salt make up approximately 20% of the world’s soils, and their extent is growing due to human-caused climate change and other anthropogenic factors (Eswar et al., 2021; Hassani et al., 2021; Batlle-Sales, 2023). According to some estimates, abiotic stressors reduce agricultural yields by half, posing a major risk to world food security (Manuel et al., 2017; Hopmans et al., 2021). The world’s population is growing at a rapid pace, which means food production must be boosted by 70% by 2050 (Kumar et al., 2022). Salinity stress reduces wheat crop productivity (Singh et al., 2023, 2024). A saline stress level of 6–8 dS m^−1^ is the threshold at which wheat crop production begins to decrease (Abbasi et al., 2016). The FAO reports that 397 million hectares of wheat crop are in danger of becoming badly salinized, posing a major risk to the global food supply (Isayenkov and Maathuis, 2019). The physiological processes of plants are disturbed, and the ultimate yield is severely reduced because of salinity stress-induced ion toxicity and nutritional imbalance (Hannachi et al., 2022). Salt stress reduces seed germination significantly at first, and thereafter it changes growth and reproductive behavior, leading to major yield losses (Munns, 2002). In addition to inducing oxidative stress, salt stress disrupts enzyme activity, photosynthesis, chloroplast structure, hormone balance, water, and nutrient absorption (Qin et al., 2020). Salinity stress, a polygenic trait regulated by multiple genes, affects plants by excluding Na^+^ and retaining K^+^, level, maintaining the K^+^/Na^+^ ratio at its ideal, osmotic adjustment, and enhancing antioxidant activity (Rana and Kalaichelvan, 2013; Allan et al., 2021; Hannachi et al., 2022). Although they take both resources and time, methods including nanotechnology, screening, genotype selection, gene introduction, and conventional breeding have been utilized to increase crop yield. Promising outcomes can be obtained through osmoprotectant use, seed priming, nutrition control, and hormone treatments.

The Food and Agriculture Organization (FAO) of the United Nations reported that ~10% of the global population faced undernourishment in 2020, affecting over 700 million people (FAO, IFAD, UNICEF, WFP and WHO, 2021). Additionally, more than 2 billion people suffer from hidden hunger caused by insufficient micronutrients and vitamins (Garg et al., 2018). A key factor is the emphasis on increasing crop yield rather than quality, leading to nutrient deficiencies in grain crops and their consumers. Despite dietary changes, including more dairy, fruits, and legumes, many still face nutritional deficiencies (Khan et al., 2020). This highlights the urgent need for a nutritional revolution to enhance crop quality, prompting agriculturists to develop nutrient-rich crops using advanced techniques (Pandey et al., 2016; Khan et al., 2020). Biofortification of key food crops can be used to improve micronutrient concentrations through a variety of approaches, including agronomic biofortification, conventional or marker-assisted selection, and genetic manipulation (Cakmak, 2008; Bouis et al., 2011; Sharma et al., 2017; White et al., 2017).

**Figure 1:**
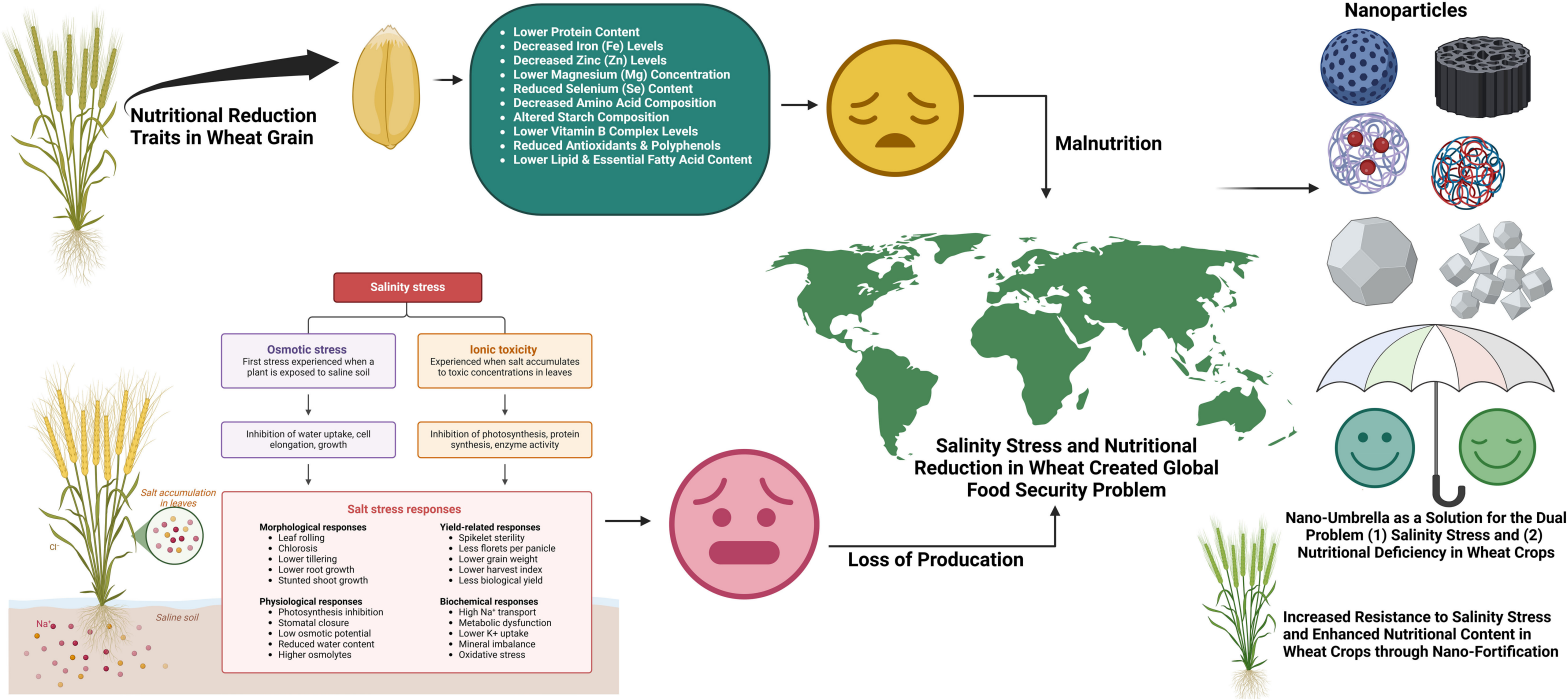
Salinity is detrimental to wheat growth by affecting osmotic imbalance, ion toxicity and oxidative stress, resulting in reduction of yield and poor quality of grains. NPs play a role of a “nano-umbrella” in increasing salt tolerance through the control of ion transport, enhancement of antioxidant defense, modulation of stress-responsive genes and osmotic adjustment. Furthermore NPs could also promote the biofortification of wheat grains with vital micronutrients involved in enhancing not only resilience against abiotic stress but also nutritional quality.

Nanotechnology is considered one of the novel approaches to enhance crop resistance to abiotic stresses like salinity (Pirzada et al., 2024). NPs have emerged as a promising, environmentally friendly, and cost-effective approach to mitigate the harmful effects of salinity stress (Taqdees et al., 2022). NPs have special physicochemical features which make them potential candidates for improving plant tolerance to salinity stress. NPs are the particles having size from 1 to100 nm possess many unique characteristics like their different shapes (e.g., spheres, rods, tubes, fibers, discs, squares), high surface area by volume ratio, crystal structure, adjustable pore size, and are involved with important activities at cellular and molecular level in living systems (Subbenaik, 2016; Sarkar et al., 2021; Taqdees et al., 2022). These are helpful activities by NPs as they are well known for agriculture benefits such as increased production, plant immunity, stress mitigation, disease management (Ragavan et al., 2017; Djanaguiraman et al., 2018). The presence of NPs causes numerous significant morphological, physiological and biochemistry effects on these plant species. NPs have been reported to mediated salinity tolerance in different plants via altering hormonal levels, antioxidant enzymes, ion homeostasis, expression of specific genes and defense mechanisms (Wahid et al., 2020; Zulfiqar and Ashraf, 2021). Earlier, metal oxide NPs like FeO, CeO_2_, Al_2_O_3_, TiO_2_ and ZnO have been used in the agricultural sector as well as their role in effective pest, disease control and toxicity along with improved cereal yield under abiotic stresses (Zheng et al., 2016). Nowadays use of NPs in agriculture requires a comprehensive information about their behavior in soil and their interactions with soil constituents. NPs based fertilizers have been reported as a solution for numerous benefits including controlled release of nutrients and enhanced water holding capacity gained through its high surface area and reactivity as identified from studies (Baruah and Dutta, 2009; Gilbertson et al., 2020). Utilization of NPs based fertilizers reduced the excessive use of chemical fertilizers, nutrient loss, enhance of crop yield with fortification quality (Baruah and Dutta, 2009; Prasad et al., 2017; Singh et al., 2017; Mohammed et al., 2020). Consider all these aspect NPs can act as a protective umbrella with two dual role (a) protection against salinity stress with (b) increasing the nutrition into wheat grain (**Figure 1**).

The objective of this review is to deliver useful information and trends of NPs research in alleviating salinity stress and enhancement of biofortification in wheat crops. In detail this work is focus on NPs based sustainable approaches to improve the, physiological, biochemical, and molecular traits which help in productivity and quality of wheat crops under salt stress conditions also help for biofortification in wheat plants. It also highlights the current gaps in the research and suggests avenues for future research that will help move this important area of research forward.

## 2 Effect of salinity stress on wheat

New applications such as NPs-mediated management are emerging as appealing strategies to alleviate salinity stress in wheat (Manzoor et al., 2021; Alharbi et al., 2023; Badshah et al., 2023; Mustafa et al., 2022; Shao et al., 2024; Zafar et al., 2024a). Biofortification of crops by means of nanofertilizers can be an alternative practice to deliver micronutrients that provide beneficial impact on human health, agriculture, and the environment (Yadav et al., 2023b). NPs have  unique structural and physiochemical properties which can enhance soil or solution nutrient uptake, improve stress tolerance, and activate physiological and biochemical processes of plants (Mustafa et al., 2021, 2022; Ahmadi-Nouraldinvand et al., 2023; Ekim et al., 2024; Zafar et al., 2024b). There has been considerable evidence of NMs protecting crops, enhancing the yield, and alleviating the adverse effects of pesticides and fertilizers on cereal crops, fruit, and vegetable crops (Parra-Torrejón et al., 2023; Carmona et al., 2024). The positive perception of NPs as soil amendments to enhance biofortification in the recent eight years looks positive for the future. According to the various scientific results, Zn, the macronutrient Fe, the micronutrient Se, nanocomposites, and metalloid NPs biofortified several plant species with macronutrients and essential ingredients (Parra-Torrejón et al., 2023; Ahmad et al., 2024; Carmona et al., 2024). NPs uses also increased physiological performances, antioxidant compounds, and yields (Khan et al., 2021; Parra-Torrejón et al., 2023).

### 2.1 Growth and development of plants

**Figure 2:**
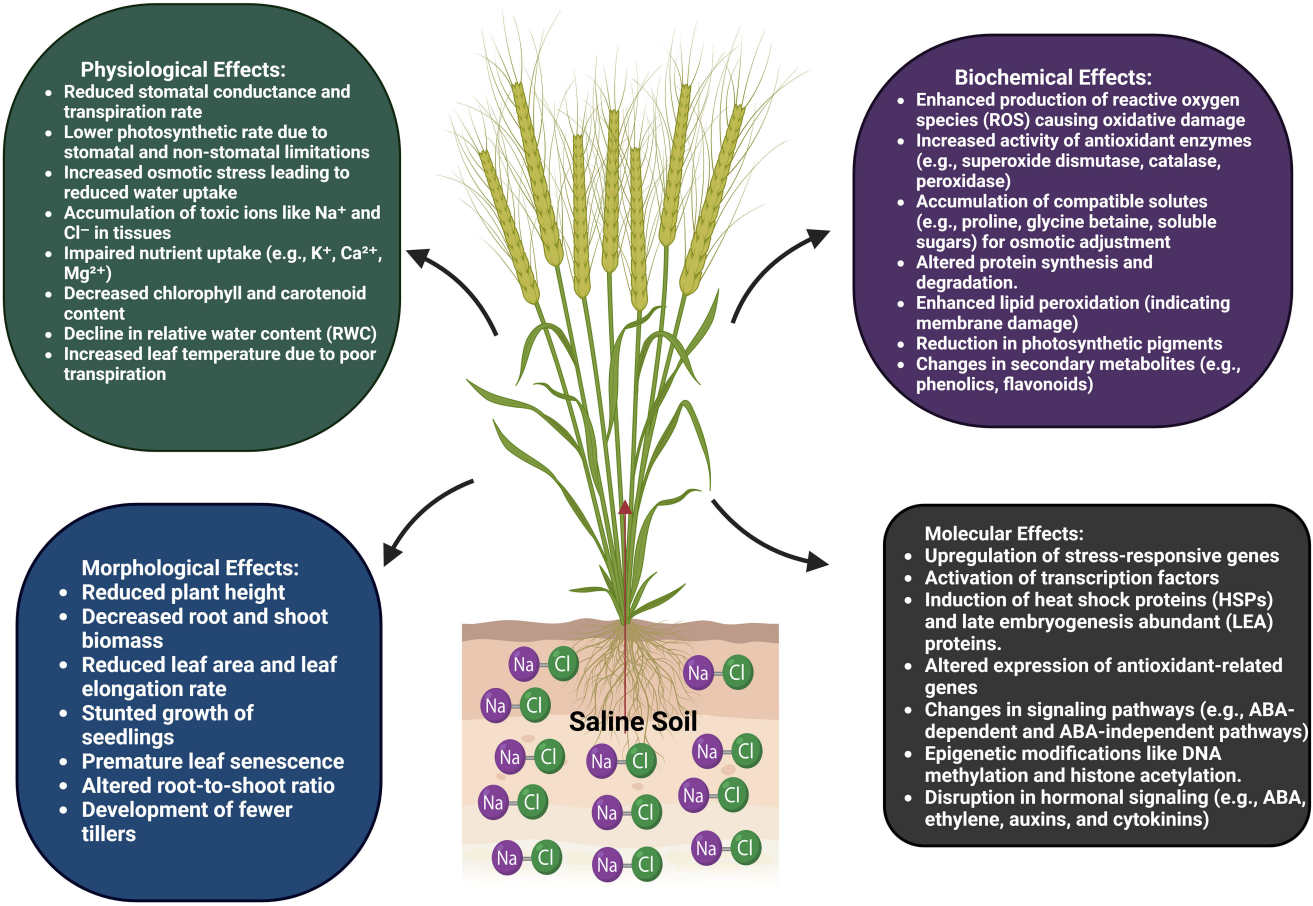
Salinity stress affected the wheat by caused alteration in physiological, morphological, biochemical, and molecular traits. At morphological and physiological level alteration show in root and shoot structure, reduced transpiration rate, closure of stomata, less water uptake, accumulation of ions. Whereas at biochemical and molecular level; lipid peroxidation, ROS production, activation of antioxidant and non-antioxidant enzymatic system, epigenetic change, activation of signaling pathways.

Plant species, stage of development, and salt concentration are among the variables that determine the rate of growth suppression caused by salt stress, which is like other abiotic stresses (Rajput et al., 2024). As a survival mechanism, stunted growth helps plants deal with salt stress (Zulfiqar et al., 2019). The decreased meristem cell counts and growth inhibition from salt stress impact a plant’s ability to absorb water and nutrients. These factors can also affect the expression of key regulatory genes involved in cell cycle development, like cyclin and cyclin-dependent kinase. When exposed to salt stress, certain plants experience “anxiety,” react rapidly, and eventually cease growth. Although some species adapt well to salt stress, others do not, and they risk extinction if they continue to grow in such environments (Adisa et al., 2019). In the plant life cycle, germination plays a crucial role in determining the features of growth, development, and yield. **Figure 2** shows that salinity stress significantly lowered wheat crop yields by decreasing seed germination. Final stand establishment and yield are both diminished by salt stress, which lowers osmotic potential and interferes with the normal functioning of enzymes required for metabolic activity (Savci, 2012). In addition to lowering biomass yield, salt stress also lowers spikelet quantity, productive tiller weight, and grain weight. Salt stress is another cause of seedling mortality because of the vulnerability of plant seedlings to stress (Sarmast et al., 2018). The parameters of both roots and shoots are adversely affected by salt stress. When wheat was subjected to salt stress, Guo et al. found that its growth was significantly slower than that under normal circumstances (Guo et al., 2015). Under the same conditions, when plants were exposed to 100 mM NaCl, both root and shoot lengths, as well as their dry weight, decreased (Zou et al., 2016). The yield of nearly all crops is drastically reduced by salinity stress. Nevertheless, salt-tolerant, and sensitive types may experience different percentages of yield decline.

### 2.2 Nutrient toxicities and imbalances

Reduced nutrient and water intake is one negative consequence of being in a salty environment, particularly one with a high salt concentration in the soil solution. Because of this, osmotic stress worsens ion toxicity and nutritional imbalance in situations where water is scarce. Cereal leaves are damaged by salinity, which causes chlorosis and triggers leaf senescence (Hanin et al., 2016). Nutrient deficiencies and excesses are caused by ion combinations in salty environments (Hawkins and Lewis, 1993). Ionic imbalance’s fundamental mechanism has just lately been clarified. Salt ion transport within the vacuole tends to counteract the plasma membrane’s ion flow into the cell, preventing the nutritional imbalance at low-moderate salinity (Bajji et al., 2002; Ludwiczak et al., 2021). In cells, ionic homeostasis is disturbed when the inflow rate is increased, leading to an imbalance in nutrients, particularly potassium and phosphorus, as anions (Cl^−^) and cations (Na^+^, Mg^2+^, Ca^2+^) build up in the cell’s plasmatic compartments (cytosol, matrix, and stroma) rather than the vacuole. Ion accumulation in the apoplast persists, and symplast ion transit does not happen in the short term, so that ion imbalance is avoided. The ionic imbalance caused by salinity is typically thought to be caused by the buildup of these ions in the apoplast (Grattan and Grieve, 1998). Salt builds up in the apoplast of leaves, which causes a disruption in the water connections between cells and eventually wilting (Flowers, 2004; Flowers and Colmer, 2015). The “generalized dose-response curve” and the concentrations of vital nutrients in the root media affect plant growth in saline environments. However, plant growth can be hindered by nutrient-induced deficit or toxicity when conditions are not ideal (Zhang et al., 2010). The reduction in nutrient availability resulting from competition with major ions (Na^+^ and Cl^−^) is the reason for the difficulty in acquiring minerals in salt stress conditions often resulting in deficiencies in Ca^2+^, K^+^, and Mg^2+^. There is a complicated link between salt stress and important mineral elements including potassium, phosphorus, and nitrogen (Singh et al., 2022a). Nitrogen is essential for plant cellular components, while phosphorous is required for photosynthesis, storage, and energy transfer. Potassium is vital for protein synthesis and water relations, and the cellular balance between sodium and potassium is essential for plant survival.

### 2.3 Plant water relations imbalances

The water status of plants is difficult to measure since it fluctuates minute-to-minute. In the near term, it relies solely on stomatal conductance, and it might be challenging to get reliable values using psychometric or pressure chamber measurements (Zhu et al., 2018). An improvement in salt tolerance would enable plants to extract more water from low-rainfall regions where salt lingers in the subsoil (Munns, 2002). Most wheat yield losses happen during heading time because of water stress, and the losses are even worse following anthesis (Hasanuzzaman et al., 2011; Zheng et al., 2012; Kuznetsov and Alamer, 2023). Rapid ion absorption causes ion buildup in plant cells, which in turn disrupts the equilibrium between plants and water. When plants are subjected to salinity stress, their turgor pressure drops and their water-holding capacity diminishes because an osmotic gradient is formed by the soil’s high salt concentration (Shahid et al., 2018). Short-term salinity stress on plants reduces different growth indicators, such as relative water content, water intake, and transpiration rate (Petridis et al., 2012). Previous a study shows that durum wheat is one of salt tolerant crop species (Flagella et al., 2023). But finding of another experimental results presented that salinity stress at 200mM NaCl concentration decreases shoot dried biomass of all durum wheat genotypes examined (Borrelli et al., 2018). The greatly reduced shoot chlorophyll contents in plants are linked with the toxic effect of the accumulated Na^+^ (Acosta-Motos et al., 2017). A 200 mM NaCl concentration stunted wheat shoot growth by damaging the photosynthetic machinery, causing turgor loss, stomatal closure, and reduced cell expansion and division (Borrelli et al., 2018). Under escalating salinity stress, plants maintain steady-state turgor pressure by decreasing their osmotic potential relative to total water potential (Paul, 2013; Ludwiczak et al., 2021). Under normal transpiration circumstances, water travels the xylem of the roots from the soil along an apoplastic pathway propelled by a hydrostatic pressure gradient. When salt levels are too high to allow for transpiration, however, the cell-to-cell route becomes the dominant means of water transport across membranes (Elsheery et al., 2020; Kerepesi and Galiba, 2000).

### 2.4 Energy transduction and carbon assimilation reactions

The process of photosynthesis which transforms the energy from the sun into chemical energy, is the most well-known and essential feature of plants. Environmental factors have a significant impact on a plant’s ability to maintain optimal photosynthetic activity, which is crucial for the plant’s (Archana et al., 2017; Zhao et al., 2020). The buildup of ions (Na^+^ and Cl^−^) in the chloroplast and a decrease in the plant water potential because of excessive salt stress hinder photosynthesis (De Villiers et al., 1996; Zheng et al., 2012). A study on the physiological responses of wheat to salt, it was discovered that salinity stress caused stomatal closure, reduced CO_2_ absorption, and lowered transpiration rate (Guo et al., 2015). The photosynthetic pigments in the chloroplast were also drastically diminished by salt stress (320 mM NaCl), leading to a precipitous drop in overall production (Qin et al., 2010, 2015). Photosynthesis still is the most important metabolic pathway determining the redox state in plant cells, regulatory networks in cells (Scheibe et al., 2005). Therefore, to evaluate the measurement characteristics of plants photosynthesis responses like photosynthetic efficiency based on chlorophyll fluorescence under stressful environmental conditions with their impact on plant growth and development (Kuckenberg et al., 2009; Santos et al., 2011). In a study the sensitive genotypes of wheat to salt stress, the chlorophyll index decreased when exposed to 90 mM salt concentration. Conversely, salt-tolerant wheat genotypes exhibited an increase in chlorophyll a and carotenoids under the same conditions (Masarmi et al., 2023).

**Figure 3:**
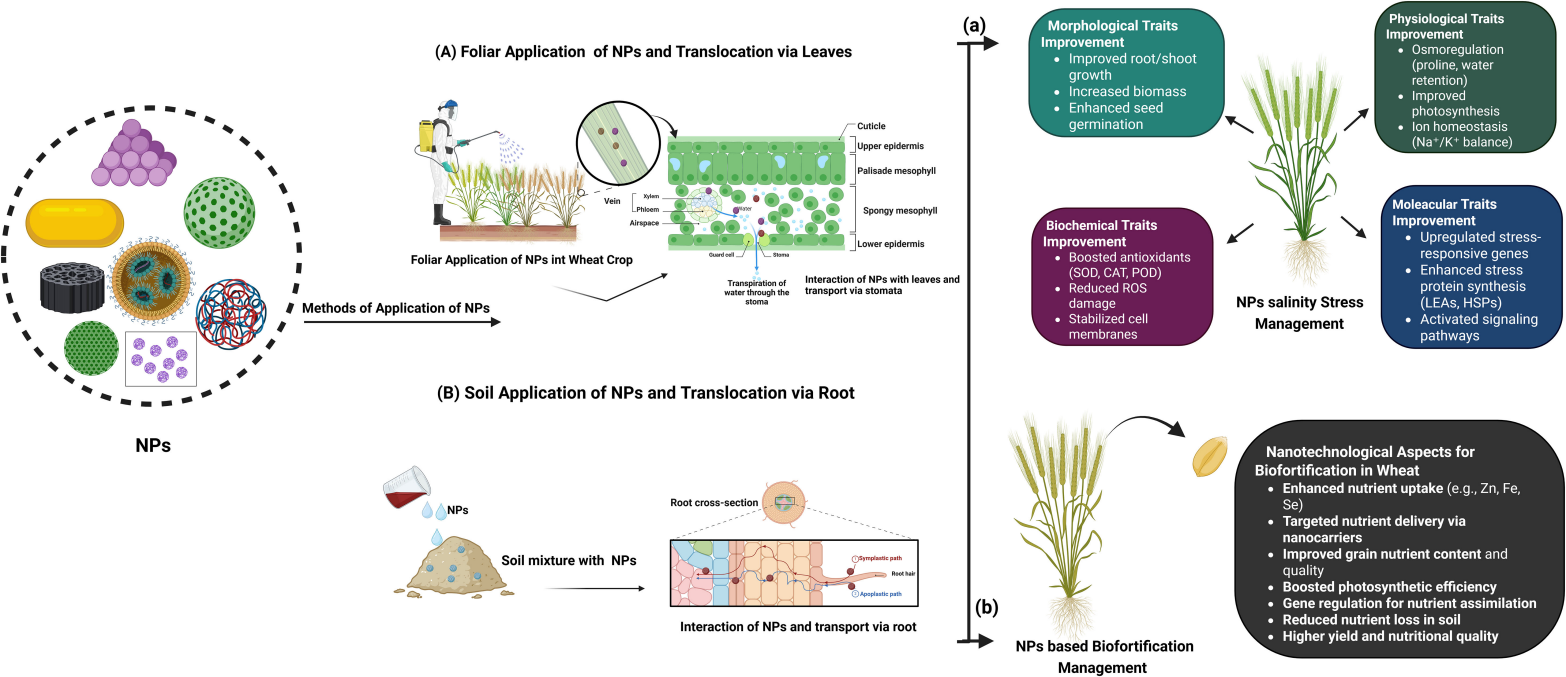
The NPs can be used either as a spray on the leaves known as **(A)** foliar application or through the **(B)** soil (absorbed by the roots). Both NPs application methods (foliar and soil) can help to improve several, **(a)** morphological, physiological, biochemical, and molecular traits and subsequently provide increased salinity tolerance to plants. Moreover, **(b)** NPs can be carried to the grains to improve their nutritive value. It enhances the nutrient quality of the grain (nanofortification), as well as the productivity of the crop and soil health. Both of these strategies render NPs a viable approach for salinity stress tolerance and the increasement of nutrient content.

A salt-induced photosynthetic reduction is associated with several variables, such as defective chlorophyll production, stomatal closure, decreased CO_2_ supply, altered enzymatic activity, and damaged photosynthetic equipment (Moradi and Ismail, 2007; Abdel Latef, 2010; Zheng et al., 2012; Tao et al., 2021). According to previous research, chlorophyll levels drop when plants are exposed to high levels of salt because the buildup of ROS causes chlorophyll to be more easily oxidized and degraded (Taïbi et al., 2016). Inhibiting the electron transport chain leads to pseudocyclic electron transport, which in turn creates an excess of ROS. Because of this, ROS changes photosystem assembly and photosynthetic proteins. Furthermore, by causing thylakoid enlargement and starch accumulation, exposure to larger concentrations of salt for shorter periods of time disrupts the dynamics of the chloroplast ultrastructure. Extensive research on the effects of salts on wheat photosynthesis has consistently shown that low salt concentrations cause photosynthetic rates to decrease, while moderate to high salt concentrations cause significant damage to chloroplast structures and photosynthetic machinery.

### 2.5 Impact on yield attributes

Salt inhibits not only those mentioned elements of plant development but also protein synthesis, energy metabolism, and cell signaling. As a result, it reduces agricultural productivity since plants need to devote a lot of energy to adapt, maintain their growth, and respond to stress, which lowers their yield overall (Munns and Gilliham, 2015). The decrease in biomass yield and the severity of the subsequent damage to the membrane are determined, respectively, by the rate of salt absorption and salt-induced osmotic stress (Volkmar et al., 2011). In 1977, Maas Hoffman proposed the idea of determining the salinity threshold at which yield is significantly reduced (Maas and Hoffman, 1977). Gaining a thorough grasp of the far-reaching effects of salt stress on plants could help researchers enhance agricultural yields in salt-stressed environments by adjusting the salt-induced response. These changes in biochemical and physiological traits may be stage-specific and contribute to the final yield potential (Ashraf and Ashraf, 2016). For example, during anthesis, early booting, and mid-grain filling, respectively, salt reduces grain yield by 39.1%, 24.3%, and 13.4%. Anthesis and an acceleration of shoot apex growth were both brought about by salt stress, which also reduced the quantity of spikelet primordia and caused spikelet to reach the final stage of development prematurely. According to Maas and Grieve (1990), this led to a decline in wheat yield potential because there were fewer spikes and fewer kernels per spike. In a similar vein, when plants were subjected to 200 mM NaCl stress before and after anthesis, it led to a decrease in aboveground biomass, ear weight, number of grains per plant, carbon, nitrogen, and carbon-to-nitrogen ratio in grains. The decrease was more pronounced from stress applied at both stages compared to a single-stage treatment (Eroğlu et al., 2020). It has also been noted that the main reason wheat yield potential is lost during reproductive stages is because there is not enough photo-assimilates. This could be because salt stress alters gene expression during pre-anthesis and grain filling. One example is the impact on fructan accumulation and carbohydrate remobilization in grains caused by changes in sucrose 1-fructosyltransferase, sucrose, fructan-6-fructosyltransferase, and fructanexohydrolase (Sharbatkhari et al., 2016). stress condition led to a 16% decrease in total grain weight plant^−1^, as well as reductions of spike length, spike weight, filled spikelet plant−1, total spikelet plant^−1^, and test weight to 8, 3, 37, 20, and 10% respectively (Yousfi et al., 2010). Furthermore, pollen sterility, decreased assimilate production, and reduced partitioning towards economically valuable plant portions (grains) all contribute to grain weight losses during salt stress. In a similar Dadshani et al. (2019) found that salt stress was linked to Na^+^ toxicity in 151 synthetic wheat-breeding lines, resulting in a 20% drop in total kernel weight and a 6% drop in starch content.

## 3 Physiobiochemical and molecular resilience response of wheat plants under salinity stress

### 3.1 Nutritional homeostasis

An essential function, ionic homeostasis controls ion flux to keep the concentration of Na^+^ ions low and the concentration of K^+^ ions high (Hasegawa et al., 2003). For ion homeostasis there is a need for a constant membrane potential and cell volume as well as to regulate intracellular Na^+^ and K^+^ ions essential for the activity of several enzymes in the cytosol (Farooq et al., 2015). To maintain homeostasis in the face of salt stress, plants eliminate excess salt from the cytosol by means of primary and secondary active transport (Cordovilla et al., 1995; Farooq et al., 2015). They then store these positively charged ions in the plasma and tonoplast membranes (Cordovilla et al., 1995). A recent study shows that two wheat cultivars (*Triticum aestivum* L. cvs. Shandawel 1 and Sids 14) grown under 0.1, 6, and 12 dS m^−1^ salinity levels show alteration in K^+^/Na^+^, Ca^2+^/Na^+^, and Mg^2+^/Na^+^ ratios (Talaat and Hanafy, 2022). In the second study, the analysis was performed with 20 wheat genotypes having 10 sensitive and 10 tolerant to salinity at two salt concentrations (90 and 120 mM NaCl) along with control under greenhouse conditions. Under salt conditions, the activities of tolerant genotypes showed a higher level of Na^+^ and Na^+^/K^+^ compared to sensitive genotypes. In sensitive genotype, K^+^ decreased, and Na^+^ and Na^+^/K^+^ significantly increased compared to control under salt concentrations of 90 mM and 120 mM (Masarmi et al., 2023). Salt stress causes a downregulation of some K^+^ genes and an upregulation of others (Cordovilla et al., 1995). An effective strategy against ion toxicity is the compartmentalization of excess Na^+^ in the vacuole, which protects the cytoplasm from the harmful effects of these ions (Li et al., 2006). The cytosolic concentrations of Na^+^ and K^+^ varied greatly among grain crop cultivars. Several affinity-based transporters involved in K^+^/Na^+^ maintenance and K^+^ absorption is present in biological membranes and are utilized by plants (Cordovilla et al., 1995; Li et al., 2006). As an adaptive property of salt resistance, plants physiologically reject excess salts. Plants are unable to regulate stomata and accumulate K^+^ when the sodium content is high (Fortmeier and Schubert, 1995). Another important tactic for dealing with salt is to raise the concentration of Na^+^ ions in the plant’s vacuoles through the tonoplast pathway, which is propelled by the proton gradient. Through the tonoplast route, plants reduce sodium transport in the shoot by accumulating Na^+^ ions in the root vacuoles (Wakeel et al., 2011). Under saline stress, plants optimize K^+^ uptake while limiting its omission. They do this by restricting Na^+^ input and taking advantage of sodium exclusion from the cell. This process guarantees that plants may survive in environments with high salt by keeping the cytosol K^+^/Na^+^ ratio constant (Wakeel et al., 2011; Yamane et al., 2012; Singh, 2022). Plants maintain ion homeostasis using a simple but dynamic process that requires a gradient that is energetically expensive to absorb necessary ions and excrete harmful ones. All kinds of plants are hazardous when their cytoplasm has higher concentrations of Na^+^. Glutamate receptors (GLRs) and cyclic nucleotide-gated channels (CNGCs) are examples of nonselective cation channels that likely facilitate sodium uptake in plants at the root-soil interface. Plants also use aquaporins and high-affinity potassium transporters (HKTs) for Na^+^ absorption (Demidchik and Maathuis, 2007; Byrt et al., 2017). Sodium ions are transported from roots to shoots via the apoplastic pathway. They pass through the root epidermis symplast and are then loaded into the xylem’s tracheid. Ultimately, they reach the shoots, and particularly the leaf blades, where they have the greatest impact (Munns and Tester, 2008). To shield their cytoplasm from the harmful effects of Na^+^, plants have evolved a variety of mechanisms, such as decreasing the rate of Na^+^ entry into the cell, increasing the efficiency of Na^+^ exit from the cell, and optimizing the vacuolarization of Na^+^.

### 3.2 Mode of action of osmoprotectant

Plants have a well-known mechanism for reducing the harmful effects of osmotic stress called osmoregulation (Hasanuzzaman et al., 2011). Sugars, polyols, amino acids, and quaternary ammonium compounds are among the organic substances that plants collect, and they all work together to lower the osmotic potential (Garg and Manchanda, 2009). When it comes to controlling the plant-water relationship, osmoregulation is responsible for activating the anti-oxidant defense mechanism (Bose et al., 2014). The osmotic adjustment that occurs because of different quantities of inorganic and organic solutes differs across cultivars and species (Paul, 2013; Ludwiczak et al., 2021). Natural osmoprotectant are water-loving, light-weight, and positively charged (Pottosin et al., 2014). The salt-tolerant bean plant cultivars were less protein-heavy and had higher concentrations of proline and amino acids than the salt-sensitive ones (Bimurzayev et al., 2021). Many plants produce an excess of the quaternary ammonium molecule glycine betaine when they are stressed by salt or dryness (Zhu et al., 2022). Plant chloroplasts produce glycine betaine in response to the negative effects salinity stress, which then builds up and helps keep photosynthetic efficiency high by adjusting the thylakoid membrane’s osmotic pressure ((Alasvandyari and Desert, 2018). Transgenic methods for altering glycine betaine’s metabolic pathways have allowed for extensive research on the compound. For instance, transgenic tobacco plants have shown a considerable improvement in salt tolerance after overexpressing the betaine aldehyde dehydrogenase gene, which is derived from the halophyte plant *Suaeda liaotungensis* and encodes an enzyme that converts betaine aldehyde to betaine (Li et al., 2003). By inserting the choline oxidase gene from *Arthrobacter globiformis* into the Indica rice, a transgenic variety was created that could withstand salt stress levels as high as 150 mM. The two-step oxidation reaction catalyzed by choline oxidase, which converts choline to glycine betaine, may be responsible for the increased salt tolerance (Annunziata et al., 2019). Engineered plants were successful in enhancing salt tolerance, although other methods were also employed. An example of this is the effect of exogenous glycine betaine on common beans (*Phaseolus vulgaris* L.) on their salt tolerance: it significantly decreased Na^+^ uptake, increased K^+^ uptake, and maintained an elevated K^+^/Na^+^ ratio (Sofy et al., 2020). Another team of researchers showed that *Dalbergia odorifera* plants benefited from the exogenous addition of glycine betaine, which increased their growth rate (Cisse et al., 2021). In another study shows that higher salinity level the proline concentration in both the shoots and the roots increased that indicating a salt-induced metabolic response in wheat genotypes this shows that proline accumulation is a conserved response to salt which positive association between Na^+^: K^+^ ratio (Borrelli et al., 2018). Proline is known to play a key role in salt tolerance in wheat plants which are subject to hyperosmotic stress conditions due to salinity stress (Flagella et al., 2006; Carillo et al., 2008; Soccio et al., 2010). Two previous research studies (Szabados and Savouré, 2010; Szabados et al., 2011) did show that proline may have a protective effect on cellular structures such as membranes, proteins, and enzymes. Additionally, proline help in detoxify reactive oxygen species and act as a signal molecule to induce genes that respond to salt stress. In response to salt stress, proline levels rise; this antioxidant and essential osmolyte helps plants keep their cell turgor. The initial step in the production of proline is catalyzed by the enzyme pyrroline-5 carboxylate synthetase, or P5CS. Salinity-induced proline buildup is mediated in large part by P5CS1, one of two P5CS isoforms. Researchers have shown that *Arabidopsis thaliana* becomes extremely sensitive to salt stress when the P5CS1 gene is knocked out (Székely et al., 2008). To protect soluble enzymes (glucose, sucrose, and trehalose) from salt stress-induced toxicity caused by increased concentrations of intracellular inorganic ions, a higher buildup of soluble carbohydrates is necessary (Khedr et al., 2003).

Osmotic damage can be prevented by trehalose’s unique property, which allows for reversible water absorption capacity (Chen and Jiang, 2010). As examples of an osmolyte, we have proline, glycine betaine, salicylic acid, and sugar alcohols such as trehalose, sorbitol, and mannitol. Applying these substances externally helps improve wheat’s salt tolerance because they stabilize membranes, maintain osmotic pressure, and facilitate protein synthesis. Plants can produce more osmolytes, such as glycine, betaine, sugar alcohols, and sorbitol, when they are subjected to salt stress (Chen and Jiang, 2010).

### 3.3 Mode of action of antioxidant defense system

Reactive oxygen species (ROS), severe osmotic stress, and ion toxicity in plants are developed due to oversaturation of the root zone with salt (Mittler, 2002). Damage to membrane lipids, proteins, and nucleic acids is caused by the salt-induced accumulation of ROS, which has a significant oxidative ability and leads to irreversible metabolic failure. To neutralize ROS produced by salt, which are likely produced by the electron transport chains of chloroplasts and mitochondria, plants possess antioxidant enzymes as well as nonenzymatic compounds. Among the many types of antioxidants, some are enzymes, and others are molecules. Superoxide dismutase (SOD), ascorbate peroxidase (APX), catalase (CAT), and peroxidase (POX) are examples of such enzymes (Raza et al., 2023). Antioxidant enzymes and molecules neutralize or absorb the increased quantities of ROS, including H_2_O_2_, singlet oxygen (O_2_), hydroxyl radicals (OH^−^), and superoxide (O_2_^−^), that occur under salt-stress situations. Oxidative stress and protein breakdown are two effects of ROS in plants (McCord, 2000). By activating enzymes such as superoxide dismutase (SOD) and catalase (CAT), salt-tolerant plants establish an anti-oxidative mechanism (Mahajan and Tuteja, 2005; Fahad et al., 2014; Qin et al., 2015; Isayenkov and Maathuis, 2019; Singh et al., 2024). Numerous studies have demonstrated that in plants subjected to abiotic stress, the antioxidant defense mechanism successfully limits oxidative damage (Singh et al., 2024). There is a significant link between salt tolerance and antioxidants in certain wheat species (Zheng et al., 2012; Mustafa et al., 2022). Hydrogen peroxide (H_2_O_2_) is produced when electrons combine with oxygen molecules in the presence of superoxide radicals (Babaei et al., 2017). The regulation of intracellular H_2_O_2_ is a complex process involving multiple enzymes. Peroxidase (POD) and catalase are two of the most important of these (Singh et al., 2023). Plant cells initiate the progressive detoxification process by producing SOD. The first line of defense is SOD, which reduces hydroxyl radicals and removes superoxide radicals by turning them into oxygen and hydrogen peroxide. Hydroxyl radicals are formed when superoxide radicals convert metal ions (Fe^3+^ and Cu^2+^). These radicals can oxidize lipids and cellular membranes, causing them significant harm. Next, POX and CAT degrade hydrogen peroxides and byproducts produced by salt stress (Qamer et al., 2021).

The higher antioxidant activities are correlated with salt tolerance, according to numerous studies. As an example, a salt-tolerant variety (VA14) of Amaranthus tricolor, leaf plants exhibited an increased level of SOD, ascorbate, and APX to aid ROS detoxification, according to a recent study (Sarker and Oba, 2020). Increased synthesis of malondialdehyde (MDA) in response to salt stress indicates membrane damage. Salt-tolerant wheat varieties had lower membrane lipid peroxidation and less MDA generation when comparing different genotypes of wheat (Hussain et al., 2022). Another study found that wild-type soybeans with the peroxidase gene GsPRX9 overexpressed had better salt tolerance and antioxidant responses (Jin et al., 2019).

A well-documented phenomenon under salt stress is the formation of anthocyanins, a class of antioxidants, in plants. Since the mutant plant cannot accumulate anthocyanins when exposed to salt stress, the *Arabidopsis* gene known as anthocyanin-impaired-response-1 (air1) plays a role in salt tolerance by controlling many steps of the flavonoid and anthocyanin production pathways (Van Oosten et al., 2013). According to these findings, a wide variety of pigments, chemicals, and enzymes contribute to improving plants’ salt tolerance by reducing oxidative damage.

### 3.4 Phytohormone level regulation

Externally applied auxin, gibberellin, cytokinin, ethylene, and abscisic acid (ABA) hormones influence plant development and reduce abiotic (salt) stress. A few of these hormones, like indole acetic acid (IAA), gibberellins (GA), and cytokinins (CK), are recognized as growth promoters, while the others are called growth retardants. Auxin maintains ion homeostasis, increases wheat germination percentage, and increases shoot dry weight in salty environments (Iqbal and Ashraf, 2007). In addition, it has been found that auxin priming can reduce salt levels as high as 15 dSm^−1^ by enhancing the rate of wheat absorption and maintaining hormonal balance (Iqbal and Ashraf, 2013). However, GA priming improves plant growth and development by increasing the unit leaf surface area, which in turn boosts photosynthetic pigments; as a result, GA priming reduces the severe consequences of salt stress in wheat (Shaddad and El-Samad, 2013). Priming wheat with cytokinin increases grain yield in saline environments by increasing germination, growth, tiller number, and grain weight (Shaddad and El-Samad, 2013). Priming with ABA reduces soil salt uptake and increases chlorophyll concentrations (Iqbal and Ashraf, 2005). Siddiqui et al. (2018) found that under salt stress brassinosteroids significantly affected photosynthesis by increasing wheat’s absorbing power and photosynthetic rate. In a salt-affected environment, wheat showed a promising response to brassinosteroids as well (Eleiwa et al., 2011). An essential hormone, ABA activates an adaptive signaling cascade and controls gene expression to play a critical integrator function in the body’s response to salt stress. When exposed to salt stress, endogenous ABA levels rise rapidly, activating a kinase cascade (Chen et al., 2020). The regulation of water and osmotic balance is facilitated by the higher ABA levels, which lead to stomatal closure. One key component of the ABA signaling transduction pathways that is amplified by salt-stress-induced osmotic stress is the presence of sucrose nonfermenting 1-related protein kinases, or SnRK2s (Umezawa et al., 2009). The kinase activities of SnRK2.2/2.3/2.6 and the transcription factors ABA-responsive element (ABRE)-binding protein/ABRE-binding factor (AREB/ABF) facilitate the regulation of stomatal closure in salt-stress conditions (Cai et al., 2017). In addition to regulating ABRE-mediated transcription, these master transcription factors express the genes that are downstream targets for salt tolerance. By blocking the kinase activity of SnRK2, ABA insensitive 1 (ABI1) also mediates primary root development and negatively influences salt tolerance (Krzywińska et al., 2016). The methylerythritol 4-phosphate (MEP) pathway is responsible for the formation of ABA, which is enhanced with exposure to salt stress by upregulating the transcript levels of various genes involved in ABA biosynthesis. Enzymes produced in response to salt stress, such as zeaxanthin oxidase (ZEP), 9-cis-epoxycarotenoid (NCED), and short-chain alcohol dehydrogenase (SCAD), are crucial regulators of the ABA biosynthesis pathway (Zhao et al., 2021). Additionally, to avoid SOS2 overactivation, the Ca^2+^ and SOS pathways work in tandem with ABA signaling (Ohta et al., 2003). Hence, ABA mediates the salt-stress response in a complicated manner. To adapt to situations with high levels of salt, plants slow their growth. Auxin controls the plasticity of root development in response to salt stress. Reduced auxin signaling and, subsequently, downregulate auxin-mediated root development are caused by the downregulation of auxin-receptor-expressing genes (Transport inhibitor response 1 and auxin signaling (F-bOX), decreased polar auxin transport, and concurrently decreased auxin accumulation in the roots (Magome et al., 2008; Iglesias et al., 2014). Plants can improve their salt tolerance by slowing their growth rate by adjusting the quantities of bioactive gibberellin at specific points in their life cycle. To increase the plant’s tolerance to salt stress, it is essential to lower GA levels or GA signaling after germination. This is achieved via several genes associated with GA metabolism, including the DELLA protein SLR1, which inhibits GA signaling, and numerous others (Achard et al., 2006; Magome et al., 2008). Involved in a wide variety of plant physiological and biochemical processes, cytokinin encourages cell proliferation, differentiation, and development. Cytokinin contributes to salt-stress tolerance by self-sacrificing since it performs opposite functions in the plant’s adaptation to salt stress. One example is an increased salt tolerance caused by an overexpression of the cytokinin oxidase (CKX) enzyme, which inactivates cytokinin, or a loss of isopentenyl transferase (IPT), two essential enzymes in the cytokinin production pathway (Magome et al., 2008). The modulation of salinity responses is further influenced by ethylene signaling under salt stress, ethylene accumulates as a stress hormone and mediates multiple important biological processes. When the ethylene receptors ethylene response 1 (ETR1) and ethylene insensitive 4 (EIN4) were rendered inactive resulting salt tolerance potential was enhanced. On the other hand, salt stress hypersensitivity is caused by the loss of function in the ethylene-positive regulators EIN2 and EIN3 (Nishiyama et al., 2011; Peng et al., 2014). To keep plant development and stress responses in check, phytohormones and their complex crosstalks are essential for salinity stress signaling.

## 4 Nanotechnological aspects for management of salinity stress in wheat

**Figure 4:**
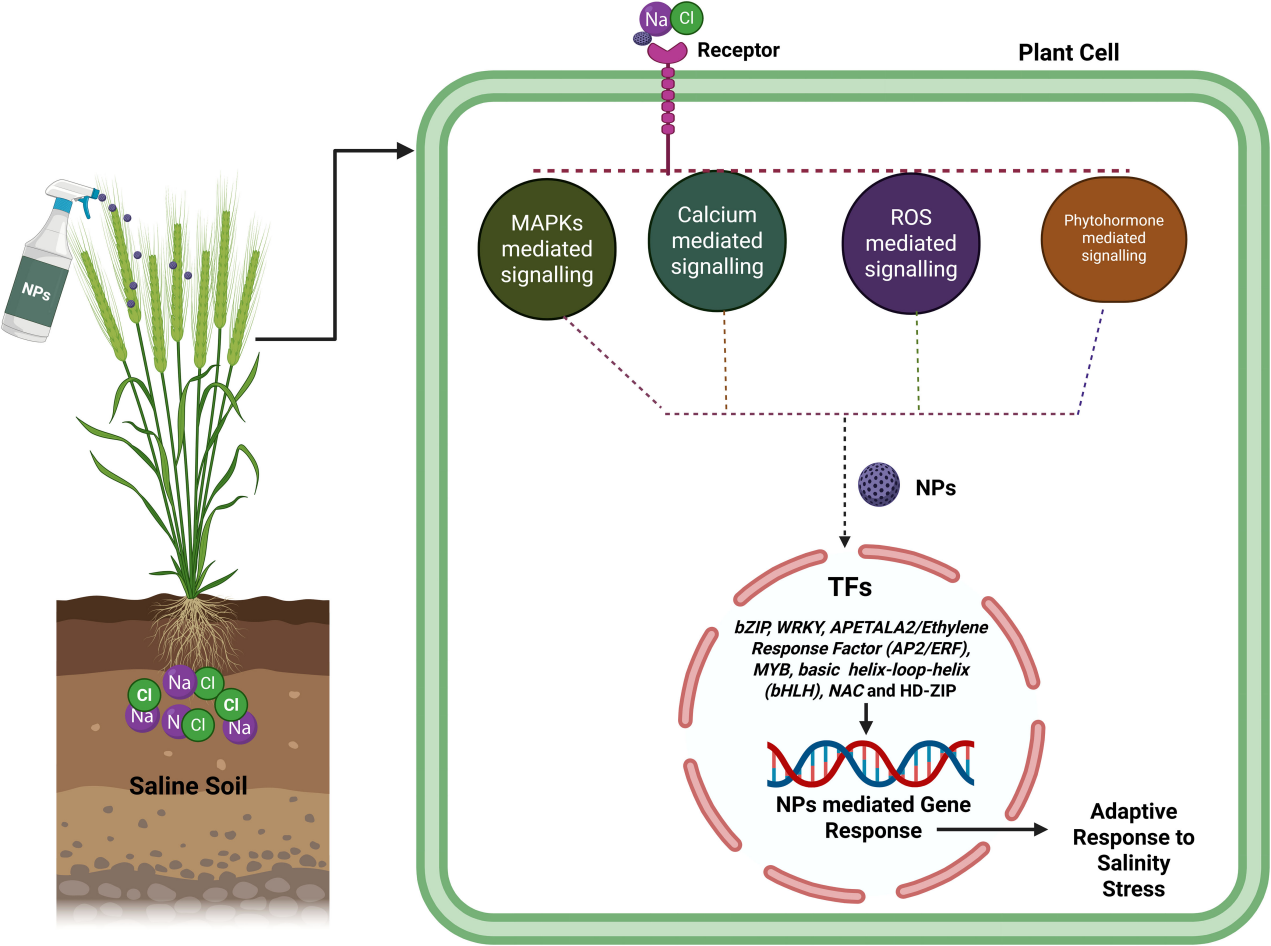
Application of NPs on wheat plant can improved in various signaling pathways like MSPKs, Calcium, ROS and phytohormone which involved in various salinity stress response TFs (*bZIP, WRKY, APETALA2/Ethylene Response Factor (AP2/ERF), MYB, basic helix-loop-helix (bHLH), NAC* and HD-ZIP) that activated or upregulated the various gene expression that participated into salinity tolerance mechanism.

In agriculture, salinity represents a key constraint for wheat production which is supported by the recently well described applications of nanotechnology in the development of novel salinity stress management strategies. **Figures 3**, **4** and **Table 1** provide more in-depth information about the recent application of NPs for managing salinity stress. NPs including ZnO, SiO_2_ and carbon-based NPs, have been show to improve wheat tolerance under salinity stress (Chen et al., 2014; Alzahrani et al., 2018; Singh et al., 2022a; Singh et al., 2022b; Ahmadi-Nouraldinvand et al., 2023; Alharbi et al., 2023; Aouz et al., 2023; Gill et al., 2024; Shao et al., 2024; Zafar et al., 2024b). These NPs help in enhancing plant morphology with root and shoot growth, increase in biomass and germination under stress conditions. At physiological level, they assist it via osmoregulation by accumulation of osmolytes such as proline, maintain balance of Na^+^ and K^+^ by conserving K^+^ and increase photosynthetic efficiency through chlorophyll accumulation and stomatal conductance (Mohamed et al., 2017; Manzoor et al., 2021; Badshah et al., 2023; Gill et al., 2024; Pirzada et al., 2024; Zafar et al., 2024b). At biochemical level, NPs counteract oxidative stress by enhancing the activity of antioxidant enzymes and stabilize cells by reducing lipid peroxidation (Ahmadi-Nouraldinvand et al., 2023; Alharbi et al., 2023; Ekim et al., 2024; Mustafa et al., 2021, 2022; Shao et al., 2024). At the molecular level, they induce expression of stress-responsive genes, ion transporter genes, antioxidant enzymes genes and transcription factors (**Figure 4**). In conclusion, nanotechnology is a valuable, sustainable tool in turning wheat against salinity for suitable production and food security in agriculture cultivated area of the world under the scenarios of climate change.

**Table 1:** Application of NPs for mitigation of salinity stress in wheat plants.

Sr. No.	Types of NPs	Application method	NPs concentration	Salinity concentration	Effects of NPs	References
1	ZnO NPs	Priming	50 mg L ^-1^	200 mM	Enhanced trapped energy flux and electron transport flux, sucrose biosynthesis AND activated the antioxidant system	(Wang et al., 2020)
2	ZnO- NPs	Foliar	0, 20, 50 and 80 mg L ^-1^	6.3 dS m1	Upregulated CAT, POD and ascorbic acid enzymes, increased chlorophyll contents, plant height, shoot lengths, root fresh, dry weights	(Lalarukh et al., 2022)
3.	Si- NPs	Soil	100 mg/kg	100 mM	Upregulated SOD, POD and CAT, Mitigated salinity stress and improve seed germination, photosynthetic pigments	(Singh et al., 2022a)
4.	Ag- NPs	Priming	0,2,5 and 10 mM	25 and 150 mM NaCl	Upregulated POD enzyme, Increased the soluble sugars and proline content	(Mohamed et al., 2017)
5.	ZnO-NPs	Priming	50, 100 and 500 mg L^−1^	150 mM NaCl	Improved wheat growth biomarkers, mitigate the effects of salt by altering growth, photosynthetic pigments, photosynthetic efficiency, and leaf ultrastructure	(Abou-Zeid et al., 2021)
6.	Si-NPs	Foliar	0, 30, and 60 mg/L	0, 35, 70, and 105 mM	Mitigating the salinity stress by enhancing antioxidant activity, improved physiological parameters by stomatal conductance, electrical conductivity, electrolytic leakage, and proline	(Ahmadi-Nouraldinvand et al., 2023)
7.	TiO_2_ NPs	Foliar	25, 50, 75, and 100 µg/mL	50, 100, and 150 mM	enhanced the biochemical attributes: free amino acids, soluble sugar content, proline content, SOD, and POD	(Badshah et al., 2023)
8.	SeNPs	foliar	0.01%, 0.05% and 0.1%	12 dS/m	scavenge ROS and protect chlorophyll from oxidative damage, enhance the activity of antioxidant enzymes, upregulate the activity of enzymes such as CAT, SOD, and POD, regulate ion homeostasis, modulate ion transporters and channels, promoting the efficient uptake and translocation of essential nutrients.	(Zafar et al., 2024b)
9.	Ag-NPs	Foliar	30, 40, and 50 μg/mL	100 mM	Enhanced seed germination efficiency, mitigated oxidative stress by inducing antioxidant enzyme, Regulated salt tolerance	(Wahid et al., 2020)
10.	Fe-NPs	Soil	100 mg kg^−1^	0.85% NaCl, *w*/*v*	Increased photosynthetic pigments, NPK, antioxidant enzyme activity, Increased plant growth, weight, and biomass, reduced the uptake of Cd by 72.5%	(Manzoor et al., 2021)
11.	Zn-NPs	Foliar	2 g L^−1^	0, 75, and 150 mM	accelerated plant height, leaf area, shoot dry weight, and iron (Fe) and zinc (Zn) concentrations	(Fathi et al., 2017)
12.	Cu-NPs	Soil	0, 25, 50 and 100 mg kg^−1^	–	Increased plant growth, biomass, and cellular antioxidants contents, decreased the ROS and Cr translocation from soil to roots	(Noman et al., 2020)
13.	Ag-NPs	Priming	1 mg L^-1^	25, 100 mM	Stimulate the germination and growth of wheat grains, improved growth index, pigment contents and chlorophyll stability index (CSI), auxins, cytokinins and ABA contents, enhances photosynthetic efficiency as well phtyohormones balance, Reduced the harmful consequences of salinity stress.	(Abou-Zeid and Ismail, 2018)
14.	Zinc ferrite (ZnFNP)	foliar	20 µM GA + 5 μM ZnFNP	–	Enhance wheat growth, potentially improve N, P, and K in both grain and shoot of wheat, Upregulate antioxidant levels in salinity, alleviation of oxidative damage and enhancement of plant resilience to salinity stress, enhance the uptake of nutrient,	(Shao et al., 2024)
15.	ZnO NPs	Priming	0, 25, 50, 75, and 100 ppm	2 and 12 dS m^−1^	improved the working of antioxidant enzymes, increased biochemical attributes, and helped regulate water relations and lipid peroxidation	(Zafar et al., 2024b)
16.	PVP–Cu NP	–	100 mg L^−1^, 400 mg L^-1^	150 mM NaCl	reduced oxidative damage by stimulating the activities of antioxidant enzymes SOD, POX, and APX, ABA regeneration and GSH redox status were maintained by triggering APX, GR, and other enzyme activities belonging to the ABA-GSH cycle	(Ekim et al., 2024)
17.	Nanocellulose (NC)	priming	0, 0.3%, and 0.6%	0.7, 5, 10, 15, and 20 dSm^-1^	improving germination percent, seedling growth, root length, root surface area and Na influx	(Sherif et al., 2024)

**Table 2:** Application of NPs for biofortification of in wheat plants.

Sr. No.	Applied Nanomaterial	Type of Application	NPs concentration	Size of NPs	Nutrient Content in Grain on Addition of NPs	References
1.	Fe-NPs	Foliar	10, 20, and 30 mg·L^−1^	40–100 nm	2% increase in plant height, 25–40% increase in seed weight, accelerated maturation period of 7 days, (1–6%) increase in protein content, 3% increase in crude fiber content, 0.9% reduction in crude fat content.	(Hassan et al., 2023)
2.	Nanochitin	Soil	0.006 g kg^−1^	143 nm	enhance the yield by 23.0% for Multi spiked wheat (MSW), 33.4% for Large spiked wheat, Increased 5% and 33.4% protein, 10.3% and 32% Fe, 22.1% and 27% Zn in MSW & LSW, respectively.	(Xue et al., 2018)
3.	ZnO-NPs	Soil	0, 19 20, 25 and 30 mg kg-1)	30 nm	56-63% increase in wheat grain yield, 9.5-54.6% increase in Zn concentration in wheat seeds, (p <0.05) increase in seed vigor,	(Yadav et al., 2023a)
4.	Nano NPK	Foliar	750:90:600 mg L^−1^	–	Increased in 27.24% protein, 19.37% increase in nitrogen, 44.11% increase in phosphorus, 12.03% increase in potassium	(Burhan and AL-Hassan, 2019)
5.	Zn-CNP	Foliar	40 mg L^−1^	-	Increased 53.3 µg g^−1^ grain zinc content	(Dapkekar et al., 2018)
6.	Fe_2_O_3_ NP	Seed Priming	25–600 ppm	–	45.7% iron increase in IITR26 and 26.8% in WL711 genotypes.	(Perea-Vélez et al., 2024)
7.	ZnO-NPs	Foliar	-	-	increased wheat grain by 15.2%, reduced 17.2% of cd concentration in whole wheat grain, increased 5.2–15.7% of crude protein in wheat grain, increased 21% of Zn content in whole wheat grain	(Lian et al., 2024)
8.	Fe_3_O_4_ NP	Foliar	5 mg L^−1^	20–30 nm	Increased 50% Fe Content in wheat seedling	(Hussain et al., 2021)
9.	ZnO NP	Foliar	40, 80, 120 ppm	-	Increased 29.4% increase in protein content	(Sheoran et al., 2021)
10.	CoFe_2_O_4_ NPs	Soil	68 mg kg^−1^	–	increased the grain yield by 52% and 21%, grain Fe concentration increased by 96% and 72%.	(Perea-Vélez et al., 2023)

### 4.1 Improving plant water relations

Plants under salt stress may have stunted growth and decreased production due to water deficiencies. Research has demonstrated that NPs enhance plant water retention by decreasing transpiration loss and increasing transpiration rate. Application of Zinc ferrite NPs under salinity stress condition can improve the water status in the wheat plant cell (Shao et al., 2024). Also increased relative water content in stressful situations may enhance tissue water status associated with better plant development under stress environments (Upadhyay et al., 2021). The various physiological characteristics of plants, including water potential, photosynthesis rates, and all growth parameters, are interconnected. A change in one aspect affects all others due to their correlation. Two wheat (*Triticum aestivum* L.) cultivars, Sids 1 and Giza 168, were grown under non-saline and saline conditions (4.7 and 9.4 dS m^−1^) resulting osmotic imbalance between soil and plants, along with ion toxicity significantly decreased wheat root and stem dry matter compared to the control treatment (Talaat and Shawky, 2014). When wheat plants were subjected to saline conditions, their relative water content (RWC) and membrane stability index (MSI) values declined due to salt reducing ion and water uptake across the plasma membrane resulting in water stress (Munns, 2002). These results indicate that in saline stress conditions, osmotic and ionic stresses decreased fresh and dry weight, RWC and MSI to inhibit wheat growth. Another study demonstrated that application of silicon NPs-based biochar (Si-BC) (control (0), 1% Si-BC1, and 2.5% Si-BC) applied under 0 and 200 mM NaCl can improve the growth and RWC of wheat but without Si-BC application saline soil substantially inhibited wheat growth and lowered RWC (Gill et al., 2024). Salinity negatively impacts the physiological parameters of both wheat varieties (Faisalabad-08 and NARC-11) when compared to the control (Mustafa et al., 2021). However, the application of plant-based titanium dioxide NPs at 40 mg/L mitigates these effects and enhances MSI (40.5% and 60.1%) and RWC (21.4% and 44.6%) at 100 mM salt stress in both wheat varieties, respectively. Similarly at 150 mM salt stress, the titanium dioxide nanoparticles improve MSI (20.6% and 40.1%) and RWC (41.2% and 17.2%) in both varieties, respectively.

### 4.2 NPs based nutrition homeostasis

Plants often experience nutrient deficiencies due to the disruption of nutrient uptake and transport processes caused by salinity stress. A solution to this problem involves the use of nanoparticles to encapsulate nutrients, allowing for controlled release and efficient delivery to plant roots. This method of targeted nutrient delivery helps to address nutrient imbalances resulting from salinity stress, thereby supporting optimal plant growth and development (Adhikari et al., 2022). One important way that NPs reduce salt stress in plants is by controlling ion transport and absorption. Cell damage and oxidative stress are outcomes of salt stress, which upsets the ion balance in plant cells by depleting vital ions like potassium (K^+^) and calcium (Ca^2+^). To increase plant growth and survival NPs can help salt-stressed cells maintain ion homeostasis. Evidence for this can be found in studies that applied Ag NPs to wheat seedlings exposed to salt stress (Wahid et al., 2020). An increase in the activity of plasma membrane H^+^-ATPase and a decrease in the activity of plasma membrane Na^+^/H^+^ antiporter, respectively, can explain the observed increase in the absorption of K^+^ and Ca^2+^ ions and the decrease in the buildup of harmful Na^+^ ions. Salt stress reduced NR, NiR, and N content in wheat plants by 30%, 26%, and 38%, respectively (Wahid et al., 2020). However, Ag-NPs and NaCl combined supplementation increased these attributes, with 60%, 109%, and 98% increased activities in wheat. NaCl treatment enhanced Na^+^ and Cl^−^ levels in wheat roots and leaves. These qualities increased by 72% and 61% in roots and 75% and 74% in leaves compared to controls. Adding Ag-NP to salt-stressed plants lowered root Na^+^ and Cl^−^ levels by 70% and 73%, respectively, and leaf Na^+^ and Cl^−^ levels by 80% and 83%, respectively, compared to NaCl-treated plants. Compared to the control, salt-stressed plants had 16% and 26% lower root and leaf K^+^ levels. Compared to salt-stressed plants, cumulative Ag-NPs +Salt treatments improved these traits by 67% and 40% (Wahid et al., 2020). In wheat plants having salinity stress with foliar applying of ZnF-NPs the amount of shoot N (25.41%, 12.54% and 45.21%), shoot P (21.41%, 12.41% and 28.70%) and shoot K (25.56%, 12.92% and 40.45%) significantly increased the amount of compared to the control. In the same manner, ZnF-NPs significantly enhanced grain N (5.08%, 3.44% and 1.85%), grain P (9.77%, 6.70% and 19.68%) and grain K (10.87%, 6.11% and 17.23%) under salinity stress in wheat plants (Shao et al., 2024). Application of 40 ppm Ag-NPs increased the leaf potassium (K), phosphorus (P), and sulfur (S) in wheat plants under salinity stress (Pirzada et al., 2024). Biochar derived from silicon nanoparticles (Si-BC control (0), 1% Si-BC1, and 2.5% Si-BC2) applied to salt-affected soil grown wheat in an environment with 200 mM NaCl increased K concentration (Gill et al., 2024). Essential nutrients including Mg and K increased after the application of Se-NPs, 0%, 0.01%, 0.05%, and 0.1% in wheat plant under salinity stress (Zafar et al., 2024a).

### 4.3 NPs based regulation antioxidant defense system

Hyperproduction of ROS by cells subjected to salt stress can damage DNA, lipids, and proteins through oxidative damage. Another way that ROS buildup can hinder development is by interfering with cellular signaling pathways. Through boosting antioxidant enzyme activity and decreasing ROS accumulation NPs can improve plants’ antioxidant defense systems. Biogenic nanoparticles are reported to regulate antioxidant responses mediating oxidative stress and plant growth (Zaeem et al., 2020). In an experiment application of Ag-NPs upregulated the biochemical-stress markers including enzymatic SOD (44%), (APX) 82%, GR (89%), and GPX (20%) and GSH up to 18% and AsA up to 26% non-enzymatic content system enhanced in wheat plant under NaCl salt stress (Wahid et al., 2020). Another experiment shows in field application of 40 ppm Ag-NPs have potential effect to reduce the level of ROS formation and lipid peroxidation process by upregulating the activity of antioxidant enzymes SOD, CAT and APX in wheat plants under 200mM of NaCl saline condition (Pirzada et al., 2024). In a pot experiment priming application of 100 μM sulfur nanoparticles (SNP) of wheat plants seed show increased activities of antioxidant CAT, POD, APX, SOD and nonantioxidant level of under salt stress NaCl (100 or 200 mM) (Saad-Allah and Ragab, 2020). While enzymatic antioxidants such as SOD, APX, GR, and GPX act as the primary line of defense during the stress-induced responses, the non-enzymatic antioxidant actors namely AsA and GSH are considered mainly as the buffering system of the plant cells (Hasanuzzaman et al., 2019). The study found that wheat exposed to 250 mM NaCl experienced high levels of electrolyte leakage, MDA, and hydrogen peroxide due to oxidative stress caused by ionic toxicity and active oxygen species (AOS). This disrupted biological membranes and macromolecules, potentially leading to wheat plant growth and death in extreme cases. While application *of Azolla aqueous extract (AAE)* enhanced the activities of osmomodulators (sugars, and proline), antioxidant enzymes (CAT, POD, APX, and PPO) on wheat under 250 mM NaCl saline condition (Al-Huqail et al., 2024). But one study also suggested that APX, POD, CAT and SOD activities were significantly increased and the minimum levels of antioxidant enzymes (APX, POD, CAT, and SOD) were measured after Si application on Akbar-2019, Subhani-2021, and Faisalabad-2008 under salinity stress condition (8 dSm^−1^) but redaction this also show reduction in EL, MDA, and H_2_O_2_ production (Aouz et al., 2023).

### 4.4 NPs based molecular response

Salinity imposes two types of stresses on plants: (a) osmotic stress which is a decrease of water uptake attributable to salinity and (b) ionic stress which is attributed to an accumulation of ions in the soil (Van Zelm et al., 2020). In the last 20 years, hundreds of ion transporters associated with the tolerance of plants to stress have been identified. Some important transporters include the Na^+^/H^+^ antiporter (NHX, on the vacuolar membrane) for Na^+^ and high-affinity K^+^ transporter (HKT) for K^+^ these two transporters are involved in the uptake, long-distance transport and redistribution of Na^+^ and K^+^, respectively. In the heterologous expression system HKT proteins are placed into two groups: one as Na^+^ transporters and second as K^+^ transporter (Horie et al., 2007). Under low external K^+^ conditions, HKT1 performs Na^+^ uptake as a Na^+^ transporter, whereas at the same time, HKT2 acts as a K^+^-Na^+^ co-transporter (Zamani Babgohari et al., 2014). HKTs transporters *Nax1* and *Nax2* were identified to play an important role associated with Na^+^ exclusion into durum wheat and *Triticum monococcum* (Zhang et al., 2024). *Nax1* encodes for *HKT1;4–A2* which controls Na^+^ unloading from the root and leaf sheath xylem, and *Nax2* encodes for *HKT1;5–A* and *HKT1;5–D* and the combined *Nax1*and *Nax2* contribute to overall 60% reduction of Na*^+^* contents in wheat leaves by acting directly or indirectly into salinity tolerance molecular mechanism (Zhang et al., 2024). Previous research showed that *HKT1;5-D*, a key Na+ transporter in wheat, plays a crucial role in below toxic levels in photosynthetic tissues by removing excess Na^+^ from xylem ducts in natural hexaploid wheat (Munns and Tester, 2008). Additionally, HKT2 is involved in regulating the absorption of Na^+^ in K^+^-deficient plants to compensate for the K^+^ deficiency, and the downregulation of *TaHKT2* (*TaHKT2;1 and TaHKT2;3*) confer a tolerance to salt stress in wheat (Saqib et al., 2008). Research has demonstrated that the *HKT1;5-D* Na^+^ transporter a critical transport for Na^+^ in wheat which is responsible for removing excess Na^+^ from the xylem channels in natural hexaploidy wheat (Munns and Tester, 2008) Additionally, *HKT2* also plays a role not only in the regulated uptake of Na^+^ in response to K^+^ deficiency in plants but also that downregulation of two *TaHKT2* (TaHKT2;1 and TaHKT2;3) gene in salt-tolerant wheat genotypes (Saqib et al., 2008). On the other hand, Na^+^/K^+^/Ca^2+^ homeostasis needs to be maintained under salt stress for this Ca^2+^ ATPase (in plasma membrane and vacuoles), NSCC, Ca^2+^/H^+^ antiporter (CAX), Vacuolar H^+^ phosphorylase (VP), H^+^-PPase and plasma membrane H^+^-ATPase pumps needed (Zhang et al., 2010).

Na^+^/H^+^ antiporters (NHXs), which are secondary ion transporters to exchange H^+^ and transfer Na^+^ or K^+^ across membrane, are essential in many developmental and stress responses like salt stress response, K^+^ homeostasis, pH homeostasis, cell expansion, cellular vesicle transport in plants (Tian et al., 2017). In salinity tolerance the SOS pathway functions more importantly in Na^+^ efflux. In this pathway, Ca^2+^ is senses the SOS3/CBL4 that after binds to serine/threonine protein kinase SOS2/CIPK24 and activates SOS2. After phosphorylation modification the SOS2-SOS3 complex triggers the activation of the plasma membrane Na^+^/H^+^ transporter SOS1/NHX7 which help in taking out Na^+^ from cells (Jadamba et al., 2020). Under salinity stress, the addition of Se-NPs results in enhanced expression of ion transporter genes such as *NHX1, CAX1, SOS1, HKT1, H+-ATPase* and AQPs (*P1P1, NIP* and *N1P1*) for the maintenance of cytosolic Na+/K+ homeostasis in wheat. Remarkably, this regulation is also upregulated the genes which encoding antioxidant enzymes including SOD, GR, MDAR, CAT, and APX, that involved into detoxification and scavenging of ROS resulting reduction of MDA content in wheat plant (Soliman et al., 2022). In another experiment application of chitosan-proline (Cs-Pro) and chitosan-glycine (Cs-Gly) NPs (0, 200, and 400 mg L⁻¹) can upregulated the *TaNHX1* gene under salt stress levels (0, 200, and 400 mM NaCl) in wheat (Gholizadeh et al., 2025). Transcription factor gene families are *bZIP, WRKY, APETALA2/Ethylene Response Factor (AP2/ERF), MYB, basic helix-loop-helix (bHLH), NAC* and HD-ZIP play important role in determining the expression level of genes involved into salt tolerant by regulating the antioxidant and phytohormone system (**Figure 4**) (Deinlein et al., 2014; Dhawi, 2023).

## 5 Nanotechnological based nutrient “biofortification” management in wheat

With the world population growing, global food demand is increasing and it is projected that by the year 2050 the population of individuals at risk of hunger will increase by 33–47% (van Dijk et al., 2021). Micronutrient deficiency or “hidden hunger” is a big issue for low-income countries who are dealing with food insecurity and food scarcity, especially in Sub-Saharan Africa (23%), the Caribbean (17%), and Southern Asia (15% in the population) (van Dijk et al., 2021). Together, Protein–energy malnutrition and micronutrient malnutrition represent one of the largest health burdens seen worldwide with millions of deaths, predominantly of pregnant women and young children (Khan et al., 2022). Moreover, micronutrient nutrition deficiencies have also been attributed to the contemporary health challenges such as overweight, obesity and poor recovery from COVID-19 this increases the economic cost of malnutrition related problems (James et al., 2021). Deficiency of micronutrients in human diets, like vitamins (vitamin A, B9) and minerals, like Fe, Zn impacted negative health implications like growth retardation, dementia, perinatal complications, and even death (Bailey et al., 2015). The most prevalent of these issues is Fe deficiency, which affects 25% of the world population (~1.6 billion) with Fe deficiency and Fe deficiency disease (McLean et al., 2009). Provided daily intake of Fe is recommended from 8–18 mg/day based on age, sex, and body weight, while the amount recommended for pregnant women is 27 mg/day. About 50% of all reported anaemia is attributable to Fe deficiency, termed iron deficiency anaemia (IDA) (Trumbo et al., 2001). Iron deficiency anaemia (IDA) is one of the most prevalent health problems seen in women owing to higher blood losses during menstruation cycle and parturition. The consequences of vitamin A deficiency include night blindness, xerophthalmia, and even corneal ulcerations (Gilbert, 2013). According to WHO during the pregnancy, vitamin A deficit is one of the most significant threats around the world, and a major but avoidable cause of childhood blindness (250–500 million), between 10 and 20% of the collective population in the underdeveloped countries (McLean et al., 2009). Iodine is an essential component of thy­roid hormones synthesis. Worldwide, there are approximately 2 billion people with iodine deficiency induced hypothyroid; iodine intake below 10–20 μg/day may result in the diseases of gout (Trumbo et al., 2001; Andersson et al., 2012; Cui et al., 2019; Hernandez et al., 2022);. Hence, to fulfil optimum nutritional needs for a healthy human being from the food consumed this food should be fortified with requisite micronutrients to limit the hidden hunger. Biofortification of food crops with key micronutrients is important to alleviate nutritional deficiencies and improve global human health. Staple food crops can be biofortified in several ways, including through selective breeding, genetic manipulation, agronomic biofortification, and other methods (Ottaway, 2008; Khush et al., 2012).

However, none of these approaches are practical for use in a real-time framework because they are either too expensive or too time-consuming. Improved nutrient utilization efficiency and targeted nutrient delivery to plants have been areas of research focused on by nanotechnologists for the past twenty years. Fertilizers manufactured at the nanoscale (1–100 nm) with unique properties larger surface area to volume ratio and surface functionalized slow or response-based release (Raliya et al., 2018; White and Gardea-Torresdey, 2018). Since nanofertilizers requires less quantity for production and application due to efficient nutrients delivery in comparison to conventional agrochemicals. NPsfacilitate crop adaptiveness, enhance the absorption of agrochemicals, minimize their loss through volatilization, and improve effectiveness in a sustainable way. Thus, approaches based on nanotechnology, together with possible reductions in the use of chemical grade pesticides and fertilizers which would greatly reduce the bad environmental and agricultural practices. Soil fertility, plant development, and micronutrient levels can all be enhanced with the help of nanofertilizers by incorporating in zeolites and chelators (Polat et al., 2004). Nanofertilizers including zinc, iron, copper, and selenium have been extensively studied for the purpose of biofortifying food crops (Reháková et al., 2004; Polat et al., 2004; Nurchi et al., 2016; Eroglu et al., 2017). Application of ZnO at contraction of 50–1000 mg L^-1^ increased the Zn content in wheat grains (Du et al., 2019). A study evaluated the effectiveness of ZnCl_2_ and ZnEDTA to that of foliar-applied ZnO-NPs and ZnO-MPs (ZnO-microparticles) in wheat using ^65^Zn radiolabeled fertilizers. At three different Zn treatment rates – 7.5 mg/L, 75 mg/L, and 750 mg/L –following study show the impact of foliar Zn fertilizers on Zn translocation, grain yield, and grain Zn content. Grain yield was unaffected by Zn treatments even at the maximum application rate. In contrast to treatments with ZnCl_2_ (3.9%), ZnO-NPs (1.6%), and ZnO-MPs (1.8%), ZnEDTA resulted in a higher rate of Zn translocation to plant tissue (6.7%). In terms of applied Zn in grains, ZnEDTA (3.3% concentration) and ZnCl_2_ (2.5% concentration) had the highest concentrations, whereas ZnO-NP grains had the lowest concentrations, with only 0.8% concentration (Doolette et al., 2020). ZnO-NPs increase zinc concentration in wheat grain from 18 mg kg^–1^ to 40 mg kg^–1^ when applied four times. Trace elements accumulate primarily in the aleurone layer and crease region. Zn concentrations in endosperm increase nearly 30-fold with significant increases in edible portions of wheat crop which suggesting that ZnO-NPs as a suitable fertilizer for improving human nutrition (Sun et al., 2020). Another experiment showed that application of 100 mg kg^−1^ of the bioengineered FeO-NPs increased Fe content in wheat plants (Manzoor et al., 2021). Iron oxide (Fe_2_O_3_) with size 20–40 nm improved the Fe content in wheat plants (Al-Amri et al., 2020). Another experiment shows that application of Fe NPs (0, 5, 10, 15, and 20 mg L^−1^) can also increase Fe content in wheat plant (Rizwan et al., 2019). **Table 2** and **Figure 3b** explain the application of different types of NPs that help in improvement of nutrition in wheat grain.

## 6 Conclusions

Salinity is an essential limiting factor for wheat and other cereal crops which has an enormous impact on both yield and nutritional quality. Recent developments in nanotechnology have emerged as an appealing, energy-efficient, and environmentally friendly approach to alleviating salt stress in crops to improve their tolerance. In particular, nanofortification (using foliar applications, soil amendments and seed priming), holds great promise for enhancing stress tolerance, nutrient acquisition, and plant vigor in salinity stress conditions. NPs can also increase bioavailability and targeted delivery of essential nutrients, reduce ionic imbalances, and modulate stress-responsive physiological pathways. Nevertheless, further studies are needed to develop optimal formulations of nanoparticles, study their interactions with soil-plant systems, and evaluate their long-term environmental consequences before exploiting these advantages. Additionally, it is necessary to have a strong monitoring framework to enable the application of NPs in agriculture efficiently and preferred way. Thus, we need a global strategy to develop sustainable nanomaterials that can be effective for applications with minimal ecological foot print. To achieve this, agronomists, material scientists and environmental researchers need to work closely so nanotechnological approaches for sustainable crop production can be improved. Implementing precision nanofortification approaches alongside green farming techniques helps to increase food availability without posing threats to ecosystem stability. In conclusion point nanotechnology is an essential part of future food security and sustainability, which can improve both the productivity and nutritional value of crops whilst preserving the ecological balance through the merger of precision nanofortification and sustainable agriculture.
